# Infliximab in Inflammatory Bowel Disease: Leveraging Physiologically Based Pharmacokinetic Modeling in the Clinical Context

**DOI:** 10.3390/biomedicines12091974

**Published:** 2024-09-01

**Authors:** Zvonimir Petric, João Gonçalves, Paulo Paixão

**Affiliations:** 1 Department of Pharmacological Sciences, Research Institute for Medicines, Faculty of Pharmacy, University of Lisbon, 1649-004 Lisbon, Portugal; 2 Biopharmaceutical and Molecular Biotechnology Unit, Research Institute for Medicines, Faculty of Pharmacy, University of Lisbon, 1649-004 Lisbon, Portugal

**Keywords:** physiologically based pharmacokinetic (PBPK) modeling, infliximab, inflammatory bowel disease (IBD), clinical pharmacology

## Abstract

In this study, a physiologically based pharmacokinetic (PBPK) modeling framework was employed to explore infliximab exposure following intravenous (5 mg/kg) and subcutaneous administration (encompassing the approved 120 mg flat-fixed dose as a switching option) in virtual adult and pediatric patients with inflammatory bowel disease (IBD). The PBPK model and corresponding simulations were conducted using the PK-Sim^®^ software platform. The PBPK simulation indicated that a 120 mg subcutaneous flat-fixed dose might not be optimal for heavier adults with IBD, suggesting the need for infliximab dose escalation. For an older virtual pediatric patient (14 years old), subcutaneous administration of a 120 mg flat-fixed dose appears to be a feasible IBD treatment option. In the final exploration scenario, the model was extended to predict hypothetical subcutaneous infliximab doses in a virtual pediatric population (6–18 years old), stratified into three weight bands (20–30 kg, 30–45 kg, and 45–70 kg), that would yield post-switch trough concentrations of infliximab comparable to those seen in adults with the 120 mg flat-fixed subcutaneous dose. The PBPK-model-informed dose suggestions were 40 mg for the 20–30 kg band, 80 mg for the 30–45 kg band, and 120 mg for the 45–70 kg band. As demonstrated in this paper, the PBPK modeling framework can serve as a versatile tool in clinical pharmacology to investigate various clinical scenarios, such as exploring alternative dosing regimens and routes of administration, ultimately advancing IBD treatment across diverse (sub)populations of clinical interest.

## 1. Introduction

Inflammatory bowel disease (IBD) encompasses two closely related yet distinct intestinal disorders: Crohn’s disease and ulcerative colitis. The etiology and pathophysiology of IBD remain complex and multifaceted. While the exact mechanisms are still being elucidated, it is believed that a combination of genetic predisposition, environmental factors, lifestyle choices, mucosal immunity mediated by the intestinal barrier, and alterations in the gut microbiome contribute to the development of the disease and its inflammation. Infliximab, initially approved for medical use by the U.S. Food and Drug Administration agency in 1998, stands out as one of the blockbuster monoclonal antibodies (MAbs) that has reshaped the pharmacological landscape in the treatment of IBD. Specifically, infliximab binds to and neutralizes (i.e., blocks) the pleiotropic cytokine tumor necrosis factor-alpha (TNF-α), a pivotal player in the pathogenesis of IBD. Over time, or following the initiation of pharmacological treatment with infliximab, the complex interactions among (patho)physiology, immunology, and pharmacology frequently lead to a diminished clinical response. From a clinical standpoint, it has been observed that the clearance of infliximab tends to increase with the severity of the disease. Infliximab clearance is influenced by the individual’s pharmacokinetic, pharmacodynamic, and immune context. Specifically, there is a positive correlation between infliximab clearance and the extent of gut inflammation, which, in combination with immunogenicity, may lead to reduced effective drug concentrations in patients [1]. This is exemplified by the notably lower trough concentrations observed in non-responders compared to responders [2]. As a result, this exposure metric is widely employed in concentration-guided dosing approaches, encompassing dose escalation or de-escalation [1,2].

In Europe, infliximab is approved for use in both adult and pediatric populations with IBD, starting from six years of age [3]. Typically, the administration of monoclonal antibodies in pediatric patients is adapted from adult protocols. Infliximab, administered intravenously in children, follows a dosing regimen based on body weight (5 mg/kg) during both the induction and maintenance phases at designated time intervals, which is the same as in adults. However, depending on the clinical response, dose escalation or shortening the dosing intervals to less than 8 weeks might be necessary [3].

Just recently, infliximab has gained approval for subcutaneous administration as maintenance therapy in adults. This involves switching to a flat-fixed dose of 120 mg solution for injection (available in a pre-filled syringe and pre-filled pen) at week 6 following the intravenous induction phase (5 mg/kg) [4]. This innovative, subcutaneous, biosimilar infliximab can also be referred to as a biobetter, owing to its clinical novelty and improved pharmacokinetic profile compared to the intravenous originator. From a patient’s standpoint, subcutaneous administration is anticipated to enhance adherence and offer overall benefits to adult patients, resulting in better treatment effectiveness [4]. However, clinical data supporting the feasibility of switching to subcutaneous infliximab in the pediatric population is lacking at the moment [5].

### Quantitative Clinical Pharmacology Tools

Pharmacokinetic (PK) modeling, a cornerstone of pharmacometrics (the science of quantitative pharmacology) and often hailed as a state-of-the-art pharmacological tool, plays a pivotal role in clinical pharmacology. It assists in understanding drug behavior within the body, optimizing dosing regimens, and predicting drug exposure across diverse scenarios. A PK model can be conceived as a simplified empirical representation of biological processes, including drug input, distribution, and elimination, which are the fundamental aspects of pharmacokinetics within the human body [6]. However, when such an empirical model describing the fate of a drug in the body is enriched with the physical, (patho)physiological, and biochemical context of a living system, mechanistic physiological models are obtained. These models can depict physiological and biochemical processes within a virtual human. In addition to describing the pharmacokinetics of a drug, these models aid in understanding the drug’s fate within the body. Such models also have the capability to describe virtual human characteristics by utilizing known or extrapolated physiological parameters [7,8,9]. In some cases, if certain physiological parameters are not known, their values can be determined by fitting the simulation predictions to experimental data, such as the drug concentration–time profile. Namely, because of the physiological foundation, most parameters in such models are not reliant on specific drug knowledge or pharmacokinetic measurements [9].

**Figure 1:**
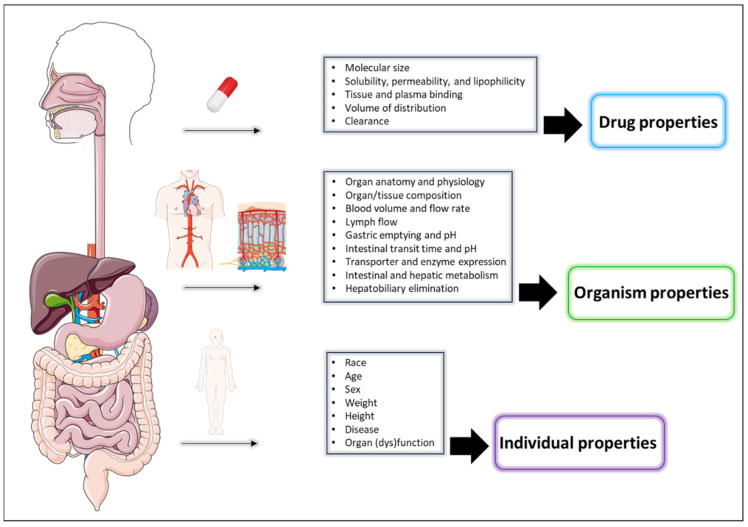
Example of integrated components of the PBPK modeling framework (graphics are with permission from Servier Medical Art by Servier, licensed under CC BY 4.0 [10]). Note that the selection of drug properties depends on the classification of the drug as either a small molecule or large molecule (e.g., MAb).

Drug-specific properties, encompassing physicochemical and biochemical characteristics, are pivotal in determining how the body processes a drug. Factors such as molecular weight, solubility, lipophilicity, and interactions with transporters—both influx and efflux—can significantly influence drug absorption. Furthermore, the disposition of small molecules may be impacted by their affinity for binding to proteins in plasma or tissue components, as well as to cytochrome P-450 (CYP) enzymes and target receptors [8]. In contrast, the disposition of large protein molecules (e.g., MAbs) is independent of CYPs, and they are mainly eliminated through catabolism. The physicochemical complexity, structural differences, and greater target selectivity and specificity of MAbs, compared to small molecule drugs, consequently affect the choice of administration route and dosing regimen. These properties ultimately manifest as clinically meaningful aspects within the realm of pharmacokinetics, such as bioavailability, volume of distribution, clearance, half-life, and various exposure metrics including the area under the concentration–time curve, maximum concentration, and trough concentration. Consequently, the framework that integrates anatomical and physiological features of a virtual individual (or population) of interest with drug-specific physicochemical and biochemical properties, utilized to characterize the pharmacokinetics of a drug, is known as physiologically based pharmacokinetic (PBPK) modeling (Figure 1). Finally, the PBPK simulation prediction, based on the corresponding model, encompasses not only the (patho)physiology of a virtual human and drug-specific properties but also the pharmacokinetic profile of the drug, its formulation, and its dosing regimen.

**Figure 2:**
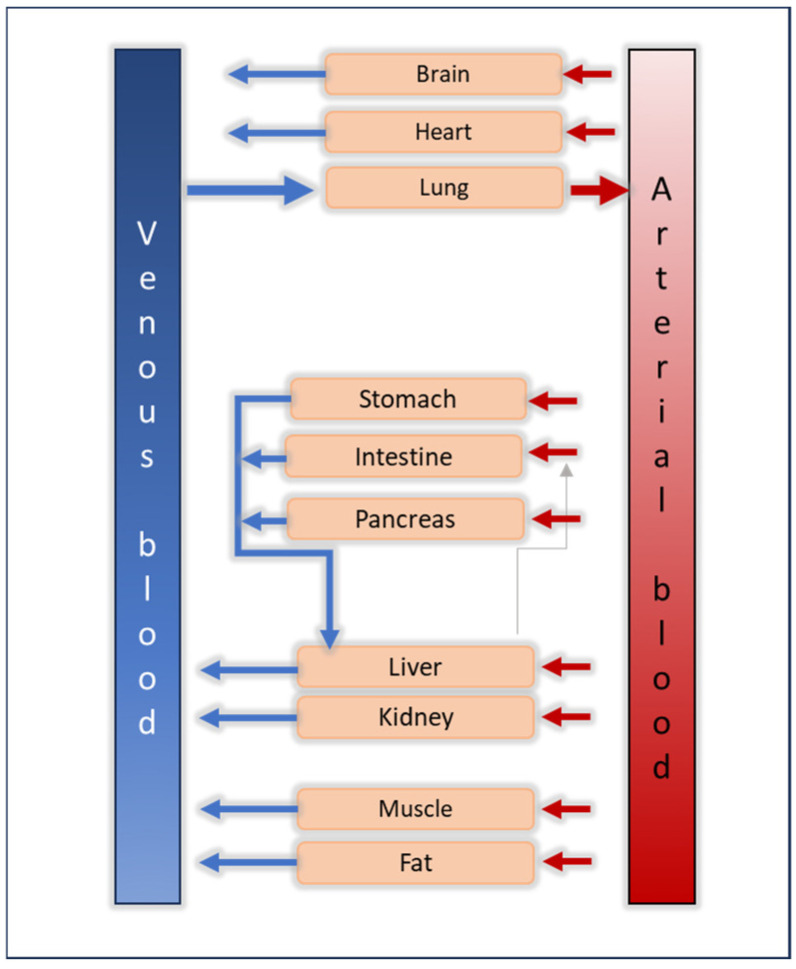
Graphical conceptualization of a PBPK model for a hypothetical drug with enterohepatic circulation. Drug input can be, for example, via the intravenous route, directly into the venous blood pool. Compartments in a PBPK model represent the actual anatomy and physiology relevant to drug input and disposition.

The fundamental assumption underlying any PBPK model is the division of the body into multiple networks of compartments, each representing organs, or part of organs, and tissues interconnected via the blood system (venous and arterial blood pool) (Figure 2) [8]. From a mathematical standpoint, the PBPK model describes the input and transfer of a drug throughout the system using mass balance differential equations. However, despite its mathematical underpinnings, PBPK modeling can offer advantages in comprehending complex clinical contexts [11], supporting drug labeling recommendations [12], or guiding dosing decisions [13], i.e., aiding in addressing questions within the realm of clinical pharmacology.

One of the primary advantages of PBPK modeling is its ability to allow users to adjust, as needed, the variability (i.e., differences) in physiological and biochemical parameters in the virtual human(s). This enables the virtual patient (or population) to reflect the characteristics of its real-life counterpart [7,8]. The versatility of the PBPK framework allows for the customization of PBPK models to accommodate various conditions such as hepatic impairment.

For instance, parameters like organ blood flow rate, liver volume, albumin and hematocrit values, and the expression profiles of CYP enzymes can be modified to mirror the pathophysiological context associated with the severity of hepatic impairment. This capability enables drawing inferences about the drug of interest, i.e., its disposition, and exposure, all of which is influenced by the impairment.

In cases where the nature of the disease is too complex to be straightforwardly set up in the software (e.g., gut inflammation in IBD), users can explore alternative methods to mimic the real-life impact of the disease on drug disposition [14]. In this paper, the versatility of the PBPK framework is demonstrated in virtual patients, including both adults and children, treated with infliximab via intravenous and subcutaneous administration. Additionally, the PBPK framework is utilized for model-informed dose suggestions in target (sub)populations of clinical interest.

## 2. Materials and Methods

The PBPK modeling exercise was performed using the modeling platform PK-Sim^®^ (version 11.2, Open Systems Pharmacology Suite, Bayer Technology Services, Leverkusen, Germany). This modeling platform offers a model specifically tailored to explain the pharmacokinetics of protein drugs and large molecules (e.g., MAbs), which is developed as an extension of a standard model for small molecules (please see the Supplementary Materials for additional information on methodology, including Figure S1) [9,15].

For large molecules and proteins, hydrodynamic radius (solute radius) is estimated from the molecular weight set in the “Basic physico-chemistry” tab. Additionally, the infliximab PBPK model included the expression profile of TNF-α (already built into the software database), with its relative expression in plasma set to 1. TNF-α half-lives in the liver and intestine were left unchanged, but its reference concentration was set to 0.20 pM [16].

PKSim^®^ does not include a subcutaneous administration building block. To compensate for this, the fat compartment was designated as the target organ for the subcutaneous delivery of infliximab, with the plasma as the target compartment. A first-order absorption process, based on the literature data [17], was assumed for modeling the absorption. The dosage modification was implemented to accurately represent the true concentration of infliximab in the biophase, taking into consideration the bioavailability (F) of subcutaneous infliximab. The bioavailability value was taken from the population PK model of the subcutaneous infliximab formulation [17]. Lastly, bioavailability (F) and the absorption rate constant (Ka) were kept consistent across all exploration scenarios involving the subcutaneous administration of infliximab.

The PBPK model of infliximab was initially developed to represent a virtual human (i.e., a virtual twin) corresponding to the average (typical), i.e., mean, pharmacokinetic, and biometric profile of the population in a real-world trial—specifically, healthy adults receiving intravenous infliximab (5 mg/kg) [18,19] (Table 1). The baseline model incorporated inputs obtained from the software, built-in parameter identification, as well as from the literature (Table 2).

The clinical trial dataset [18] in Table 1 was used for fitting this baseline model, while other trials were utilized for model validation across various clinical scenarios of interest, such as exploration of the subcutaneous administration route, different doses, or different target (sub)populations. Pharmacokinetic data on infliximab were digitized using WebPlot Digitizer^®^ (version 4.5, Pacifica, Carpinteria, CA, USA). Illustrations were made by Servier Medical Art tools under the free CC BY 4.0 license [10].

**Figure 3:**
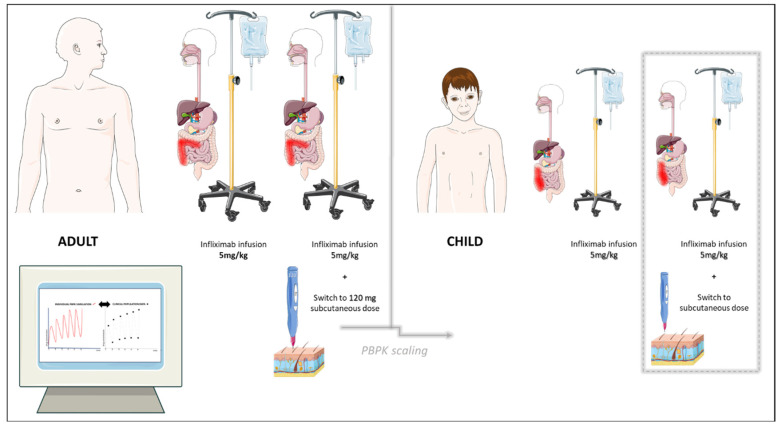
General overview of the PBPK modeling exploration of infliximab in this paper (graphics with permission from Servier Medical Art by Servier, licensed under CC BY 4.0 [10]). Pharmacokinetic profiles of a typical (mean) virtual healthy individual (70 kg), virtual adult patient (70 kg), virtual heavy adult (120 kg), and virtual pediatric patient (14-year-old child) with IBD were compared with the mean concentration–time profile derived from real-world clinical trials. The dashed line represents a hypothetical “what-if” scenario involving a switch to subcutaneous infliximab and exploration of the PBPK-model-informed dose suggestion in a typical virtual pediatric patient (14 years of age) and virtual pediatric populations (6–18 years of age) stratified by weight.

An overview of the PBPK modeling exploration of infliximab in this paper is provided in Figure 3.

## 3. Results and Discussion

This paper showcases the adaptability of the PBPK framework in patients with IBD, encompassing both adults and children, who are undergoing infliximab treatment via intravenous (5 mg/kg) and subcutaneous routes (encompassing the flat-fixed 120 mg dose). Furthermore, the PBPK model is utilized to propose model-informed dose suggestions post-switch to subcutaneous infliximab in a typical heavy adult (120 kg), a pediatric patient (14-year-old child, 60 kg), and a pediatric population (6–18 years of age stratified by weight). In essence, the PBPK simulations are employed to examine dosing strategies and to explore hypothetical clinical scenarios in target (sub)populations pertinent to infliximab dosing in the context of IBD.

### 3.1. PBPK Model Development for a Virtual Healthy Adult after Single Intravenous Administration of Infliximab (5 mg/kg)

**Figure 4:**
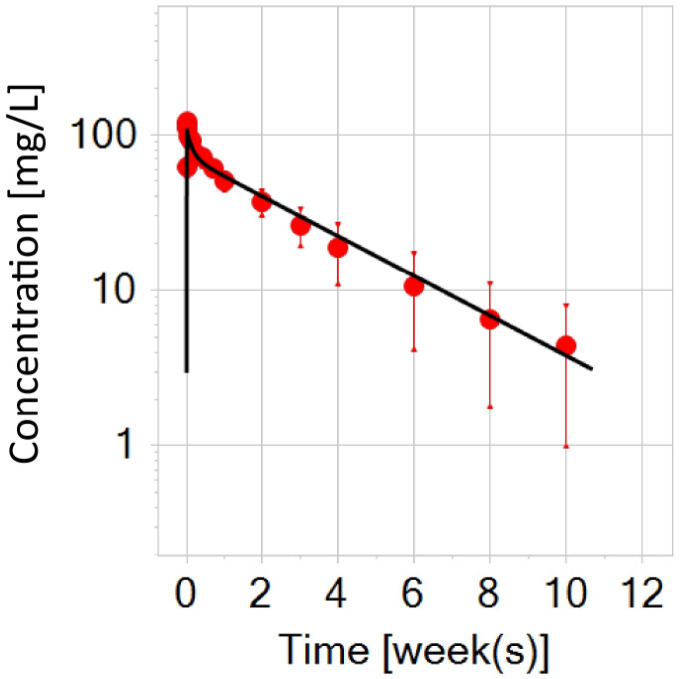
Fitted baseline PBPK model of infliximab on a semi-logarithmic scale for a typical healthy adult (black curve) after a single intravenous treatment (5 mg/kg), corresponding to a typical healthy adult from a trial. The red dots represent the average pharmacokinetic profile digitized from the trial data in healthy adult individuals.

A baseline PBPK model was constructed for a virtual healthy adult (representing a typical adult individual, 70 kg) receiving a single-dose intravenous infusion of infliximab (5 mg/kg over a duration of 2 h). The endosomal clearance rate for the typical adult healthy individual was determined by fitting the PK profiles from the trial [18] (via built-in parameter identification) to 0.91 per minute. Relevant input parameters of the baseline PBPK model are outlined in Table 2, while the fitted profile is shown in Figure 4. Trial data for a healthy individual were digitized from [18].

### 3.2. PBPK Model Development for a Virtual Adult with IBD after Intravenous Administrations of Infliximab (5 mg/kg)

**Figure 5:**
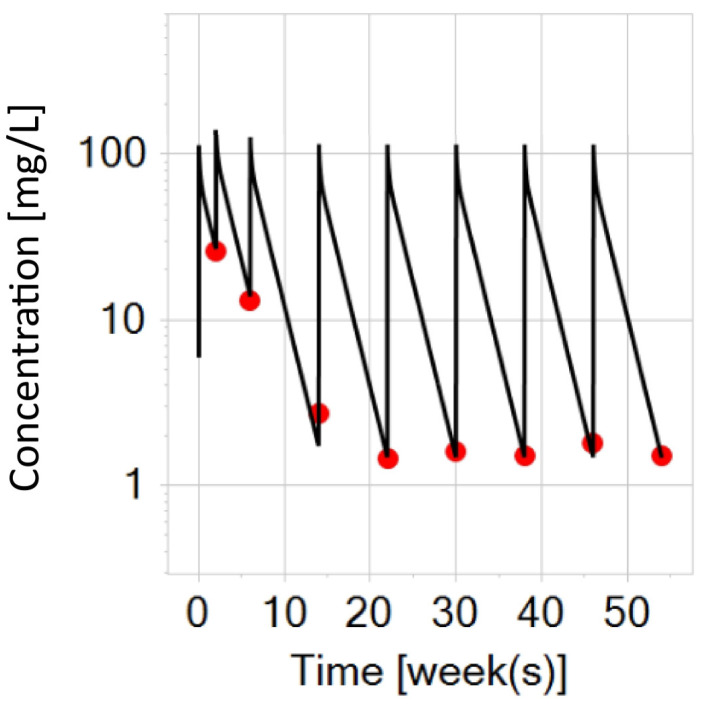
Infliximab PBPK simulation on a semi-logarithmic scale for a typical adult patient with IBD (black curve) after intravenous treatments (5 mg/kg), representing the mean adult patient from a trial. The red dots represent the mean trough concentrations digitized from the trial data in adult patients with IBD.

As previously noted, the severity of IBD can vary widely among patients, with both immune- and non-immune-mediated factors negatively impacting drug exposure. To address all the factors contributing to increased infliximab clearance, a process in the physiological compartment (i.e., endosome) was manually adjusted (as a lumped proxy for those factors) to fit an average infliximab pharmacokinetic profile in patients with IBD. Consequently, virtual adult patients with moderate to severe IBD were assigned a specific endosomal clearance rate of 1.6 per minute, which resulted in the simulation prediction shown in Figure 5. Trial data from an adult patient was digitized from [20].

### 3.3. PBPK Modeling Exploration for Virtual Adults with IBD (Typical and Heavy) after Switching to Subcutaneous Infliximab

Given the recent introduction of a biobetter of infliximab, the PBPK model was assessed to determine its capability in describing the exposure profile following the switch (at week 6) to subcutaneous infliximab at a flat-fixed dose of 120 mg in adult patients with IBD.

As stated earlier, to ensure that the actual concentration of infliximab in the biophase is properly represented in the PBPK-simulated prediction, the infliximab subcutaneous input was modeled as a first-order absorption with an estimated absorption rate constant (Ka) of 0.0114 1/h. Additionally, the dose was corrected for the estimated bioavailability (F) of 0.79. Both values were taken from the population PK study of subcutaneous infliximab [17].

**Figure 6:**
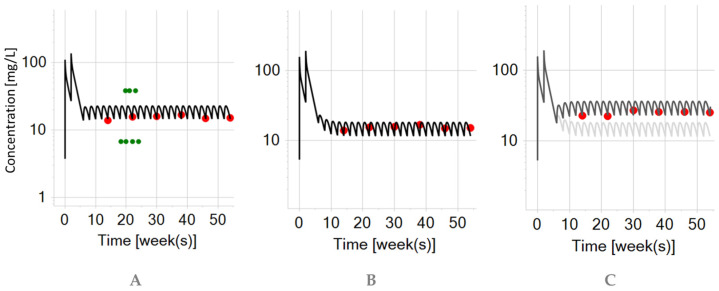
Infliximab PBPK simulation on a semi-logarithmic scale for a typical adult patient with IBD following the switch to subcutaneous infliximab (black curve) at week 6. (**A**) The image displays the prediction after a flat-fixed dose of 120 mg in a typical adult (70 kg). (**B**) The image displays the prediction after a flat-fixed dose of 120 mg in a typical heavy adult (120 kg). (**C**) The image displays the prediction after a dose of 240 mg in a typical heavy adult (120 kg). The gray curve is the prediction with a flat-fixed dose of 120 mg for the same individual (i.e., prediction (**B**)) for better visual comparison. Notice how the simulation prediction in a heavier patient (prediction (**B**)) indicates underexposure in the trough concentrations with flat-fixed (120 mg) subcutaneous infliximab dosing, while dose escalation to 240 mg (prediction (**C**)) shows a better fit to the trial data. The red dots represent digitized trough concentrations in adult patients post-switching to 120 mg subcutaneous infliximab at week 6, while the green dots represent the digitized prediction interval (5th and 95th percentile).

The PBPK simulation prediction for a typical virtual adult (70 kg) was compared to clinical data and contextualized with predicted intervals (5th and 95th percentile) derived from the population PK study [17] (Figure 6A). To evaluate the model’s predictive performance after infliximab switching (120 mg) in a typical heavy patient (120 kg), a second simulation was conducted (Figure 6B). Since there is also a clinical trial [20] which tested the subcutaneous dose of 240 mg, simulation prediction was extended to test the model performance in the typical heavy patient (120 kg) receiving 240 mg of subcutaneous infliximab (Figure 6C). It is important to mention that the subcutaneous dose of 240 mg is not approved by the regulating authority. Trial data for adult patients was digitized from [20] while the prediction interval from the population PK study was digitized from [17].

### 3.4. PBPK Modeling Exploration for an Older Virtual Child (14-Year-Old, 60 kg) with IBD after Intravenous Administrations of Infliximab (5 mg/kg)

**Figure 7:**
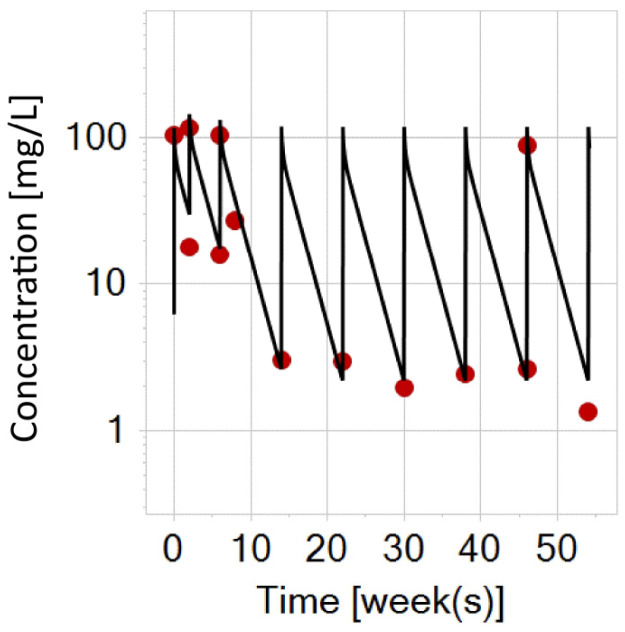
Infliximab PBPK simulation on a semi-logarithmic scale for a typical older pediatric patient (14-year-old child, 60 kg) after intravenous (5 mg/kg) administrations (black curve). The red dots represent the mean trough concentrations and maximal concentrations digitized from the trial data in pediatric patients.

To assess model prediction in the pediatric population, a virtual pediatric patient (14-year-old, 60 kg) was scaled from the adult patient with IBD. The simulation results are presented in Figure 7. Trial data from pediatric patients were sourced from [14] (originally digitized from [21]).

### 3.5. PBPK Modeling Exploration for an Older Virtual Child (14-Year-Old, 60 kg) with IBD after Switching to a Flat-Fixed Dose of Subcutaneous Infliximab (120 mg)

**Figure 8:**
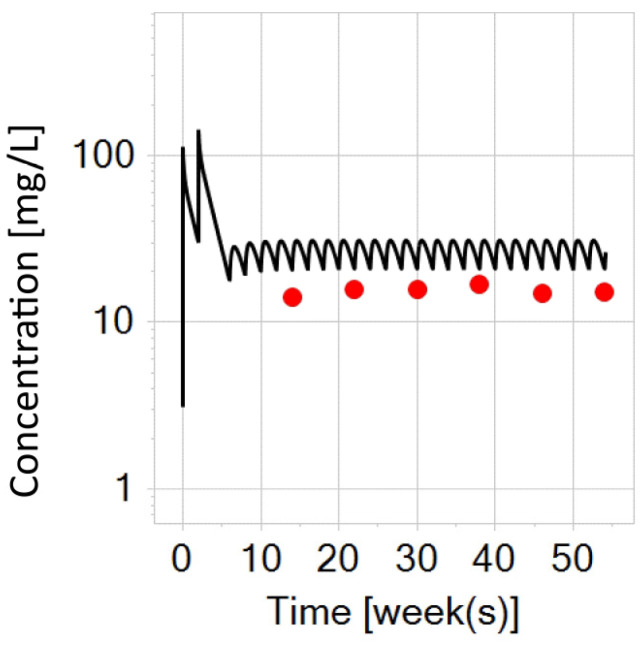
Infliximab PBPK simulation on a semi-logarithmic scale for a typical older child with IBD (14-year-old, 60 kg) after switching to a subcutaneous dose of 120 mg (black curve) at week 6. The red dots represent trough concentrations digitized from the trial data in adult patients post-switching to 120 mg subcutaneous infliximab.

Currently there is no approved subcutaneous formulation of infliximab for pediatric patients. However, older and heavier pediatric patients could have benefit from such a formulation [22]. Therefore, the infliximab PBPK model was extended to explore this route of administration in such a patient. In the absence of real-world clinical data for pediatric patients in this scenario, trough concentrations from a typical adult patient (post-switch to 120 mg) were digitized from [20] and used for comparison (Figure 8). In adult patients, median trough concentrations of infliximab post-switch to 120 mg were shown to vary between 11 and 20 mg/L, contingent on the study (see the discussion).

### 3.6. PBPK Modeling Exploration for a Virtual Pediatric Population Stratified by Weight Bands after Switching to Subcutaneous Infliximab: A PBPK-Model-Informed Dose Suggestion

**Figure 9:**
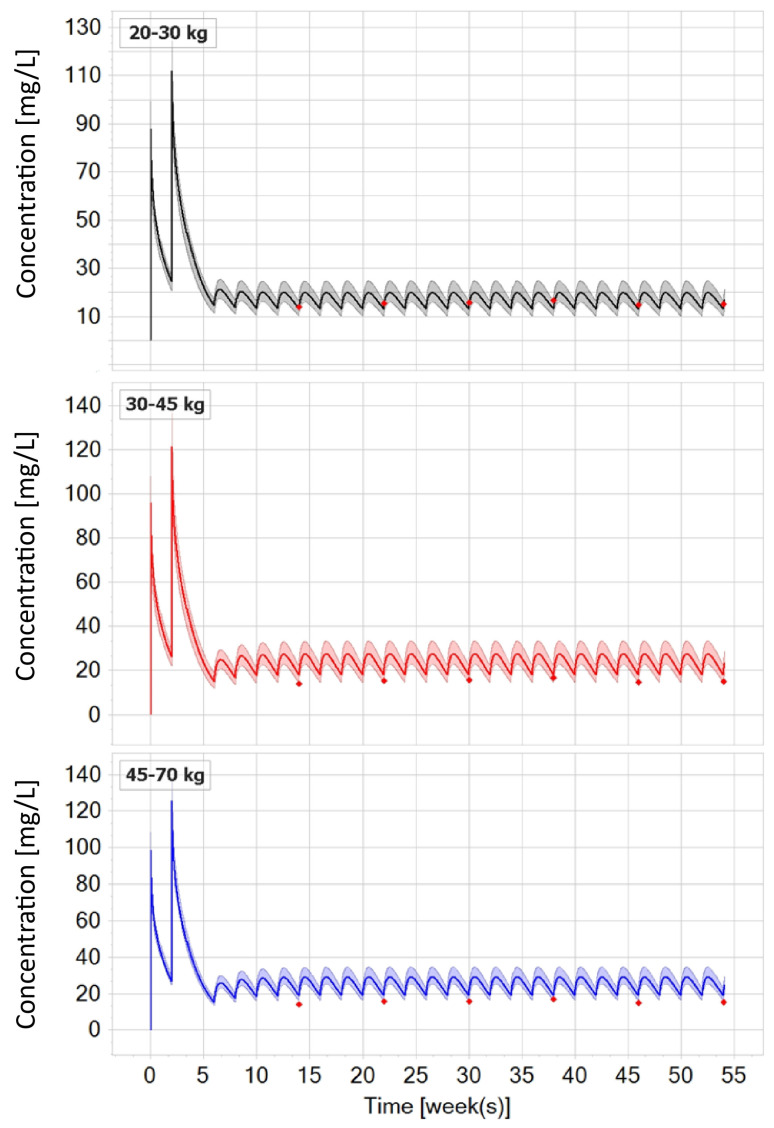
Infliximab PBPK simulations for a pediatric population stratified into three weight bands post-switch to subcutaneous infliximab at week 6. The PBPK-model-informed dose suggestion indicates that, to achieve comparable infliximab exposure metrics as in adults receiving 120 mg, pediatric patients weighing 20–30 kg would require a dose of at least 40 mg (typical pediatric patient), with a slightly higher dose needed to cover the entire prediction interval ((**top**) prediction). For patients weighing 30–45 kg, the suggested dose is 80 mg ((**middle**) prediction), while those weighing 45–70 kg would require 120 mg ((**bottom**) prediction). The red dots represent digitized trough concentrations in adult patients after switching to 120 mg subcutaneous infliximab.

In the final exploration scenario, the goal was to utilize PBPK simulations for proposing the suitable subcutaneous dose (post-switching) in the pediatric population across the approved age range for the intravenous route (6–18 years), stratified into three weight bands based on estimated weight by age [29]: 20–30 kg, 30–45 kg, and 45–70 kg (Figure 9, top, middle, and bottom image, respectively). Each weight band contained 50 simulated pediatric patients (i.e., 150 in total across the simulated age ranges). As already mentioned, given the absence of real-world clinical data for pediatric patients, trough concentrations from a typical adult patient after switching to 120 mg were used as the target exposure metric that would guide the choice of a corresponding subcutaneous dose for each weight band (if such administration were to exist). Figure 9 shows the median concentration and simulated prediction interval (5th–95th percentile) for each weight band along with the corresponding PBPK-model-informed dose suggestion: 40 mg (top prediction), 80 mg (middle prediction), and 120 mg (bottom prediction).

For the virtual healthy individual (Figure 4), overlaying the mean pharmacokinetic profile from trial [18] demonstrates that the PBPK model is able to capture the average exposure of infliximab after a single intravenous administration (5 mg/kg). This was further confirmed through a comparison of exposure metrics and pharmacokinetic parameters between a typical virtual heathy individual and real-life healthy trial participant. Namely, the area under the concentration–time curve (after a single 5 mg/kg dose) was quite comparable, totaling 40,311 mg × h/L in the simulation, compared to 38,000 mg × h/L observed in the clinical trial. Similarly, the simulated maximum concentration reached 110 mg/L, while in the trial it peaked at 121 mg/L. Additionally, the simulated infliximab clearance and apparent volume of distribution (0.009 L/h and 0.07 L/kg, respectively) were also comparable with the average dataset-derived PK parameters (0.01 L/h and 0.09 L/kg, respectively). The exposure metrics and PK parameters derived from the simulation prediction also closely match those from other publications [30]. Lastly, the baseline prediction was also plotted against trial observations, demonstrating an even distribution around the line of identity.

Pharmacological treatments with MAbs (and some small molecules) often exhibit a phenomenon where the drug’s pharmacodynamics influences its pharmacokinetics. This contrasts with the more common situation where pharmacokinetics governs pharmacodynamics, i.e., drug effect [1]. This intricate bidirectional interplay can stem from dose-dependent nonlinear elimination, termed target-mediated drug disposition, wherein drug–target complexes may impact drug effectiveness. Furthermore, the high inflammatory burden in the context of IBD triggers other elimination mechanisms, further contributing to underexposure and loss of effect, such as drug degradation via metalloproteinases [31] or excessive protein loss (which includes the elimination of MAbs) due to the leaky gut [1]. Finally, the formation of neutralizing and non-neutralizing anti-drug antibodies (i.e., immunogenicity) further contributes to underexposure (or vice versa), affecting both pharmacokinetics and pharmacodynamics. Hence, when these processes converge, they can result in unpredictable clinical outcomes and varying treatment success rates, due to faster drug elimination than expected. Therefore, adjusting the endosomal clearance rate in virtual patients with IBD to mimic the observed clinical scenario (i.e., increased infliximab clearance and underexposure when compared to healthy adults), served as a proxy accounting for the multitude of immune-mediated and non-immune-mediated pathways.

In the pharmacokinetic profile, the adult virtual IBD patient exhibited 1.5 times faster infliximab clearance compared to the clinically observed clearance in a healthy individual (0.015 L/h vs. 0.01 L/h, respectively), which aligns well with the true clinical IBD scenario after intravenous administration (5 mg/kg) (Figure 5).

The PBPK simulation of the virtual adult with IBD, after intravenous administration of infliximab, indicated a half-life of infliximab of 9.93 days, which is consistent with the clinically reported infliximab half-life of 9.5 days (with 5 mg/kg dosing) [32]. The maximal predicted concentration reached 115.5 mg/L in the maintenance phase, compared to a median of 120 mg/L in the trial dataset. Additionally, the simulation predicted a trough concentration of 1.5 mg/L (week 22), while the real-word data [33] reported a mean trough concentration of 2.9 mg/L (±2.6), i.e., adjusted geometric least squares mean of 1.8 mg/L. At week 30, Schreiber et al. [34] reported a median trough concentration of 2.3 mg/L (with the predicted interval 5th–95th percentile: 0.1–8.6). Finally, examining three larger study comparisons (C0168T72, REACH, and ACT) [35], the reported median maximal concentrations during the induction phase (with 5 mg/kg) were 115 mg/L, 109 mg/L, and 132 mg/L. In the same report [35], the median trough concentrations at week 30 were reported as 1.9 mg/L, 1.8 mg/L, and 2.5 mg/L. Since the simulated prediction of the virtual adult with IBD represents a mean virtual human, reflecting the mean of all adult real-world patients from the trial, the general conclusion, based on previously discussed exposure metrics and PK parameters, is that the PBPK model prediction in Figure 5 can effectively capture the overall trend of data in a typical adult patient with IBD after intravenous (5 mg/kg) administrations of infliximab.

PKSim^®^ lacks the subcutaneous administration option in the administration building block. As a simplification, the fat compartment was chosen as the target organ for subcutaneous input with the plasma as the target compartment. In modeling the input from the subcutaneous formulation, a first-order process was assumed according to the literature data [36], while the half-life of absorption was derived from the estimated absorption rate constant [17]. The PBPK simulation following the switch to 120 mg subcutaneous infliximab at week 6 (Figure 6, prediction A) indicates that the model effectively captured trough concentrations in a virtual adult patient (70 kg) with IBD. Moreover, simulation for a virtual heavier adult patient (120 kg) with IBD also indicates underexposure trends in trough concentrations with a 120 mg flat-fixed dose (Figure 6B). This suggests that heavier individuals with IBD could benefit from dose escalation. To test this, a typical heavy patient weighing 120 kg was compared with the real-word trial [20] encompassing a subcutaneous dose of 240 mg, which resulted in better overlapping of the mean trend of trough concentrations (Figure 6C).

These indications of a possible need for dose escalation in heavier subjects align with the conclusions drawn from the population PK study conducted by Hanzel et al. [17]. However, as subcutaneous infliximab is currently only approved in a flat-fixed dose of 120 mg, dose adjustments after switching are not expected to occur (for now).

When examining the reported trough concentrations of infliximab from various clinical trials after switching, slight variations can be expected. For instance, at week 8, Roblin et al. [37] (taken from [38]) report a median trough of 11 mg/L (with an interquartile range: 7.5–15.1), while Falquina et al. [39] report 14.1 mg/L (with an interquartile range: 12.2–22.7). In the simulation in Figure 6, prediction A, the typical adult patient had a trough concentration around 14 mg/L (at week 8), which indicates that the model is able to capture the general trend. Schreiber et al. [34] at week 30 reported a median trough concentration of 13.27 mg/L (with a predicted interval 5th–95th percentile: 5.6–26.8) for 120 mg dosing, while for 240 mg dosing, the reported median was 26.54 mg/L (with a predicted interval 5th–95th percentile: 11.2–53.2). In the prediction for the 240 mg regimen (Figure 6, prediction C), a typical heavy patient had a trough concentration of 23 mg/L. The same study [34] reported the steady state median maximum concentration (at weeks 22–30) for the 120 mg regimen to be 18.24 mg/L (with a predicted interval 5th–95th percentile: 10.14–32.63). This also aligns well with model prediction in Figure 6A, where the maximum concentration for a typical adult patient was 22.8 mg/L for the same regimen. For the 240 mg regimen (Figure 6, prediction C), the same exposure metric was around 36 mg/L for the heavy adult, while the reported value from the trial was 36.48 mg/L (with a predicted interval 5th–95th percentile: 20.3–65.15). Additionally, the PBPK model estimated half-life as 10.97 days and a clearance of 0.013 L/h (for a 120 mg flat-fixed dose). These values are comparable to those estimated by population PK modeling in a typical adult IBD patient by Hanzel et al. [17] of 10.8 days and 0.015 L/h, respectively. Hence, it can be inferred that the PBPK simulation was able to capture the general trend of the data in adult patients with IBD after switching to 120 mg flat-fixed subcutaneous infliximab dose (Figure 6A).

It is also worth reiterating that the PBPK model indicated underexposure in heavier patients with 120 mg flat-fixed dosing (Figure 6B). Therefore, additional clinical trials are warranted to validate this simulation finding and ascertain the necessity of dose adjustments for heavier adults.

When determining the dose of MAbs in pediatric patients, it is common to employ weight-based dosing (i.e., mg/kg) extrapolated from adult dosing protocols. However, this method fails to consider the maturation process, as well as the (patho)physiological and biochemical differences between these two populations, relying solely on changes in size [14,19,40]. Therefore, in cases of such extrapolation, younger pediatric patients (infants) are often underdosed.

PBPK modeling, with its mechanistic approach, provides an additional opportunity, especially when combined with allometric scaling, to refine pediatric trial design and determine better dosing strategies for pediatric patients.

In Figure 7, it seems that the PBPK simulation for the intravenous administration (5 mg/kg) slightly better captured the maximum concentrations of the induction phase in the typical pediatric patient (14-year-old child, 60 kg) which was also noted by other researchers [14]. However, variable disease-mediated influences on drug elimination over time must be also taken into account when interpreting trough concentrations in the context of PBPK modeling. In the same virtual pediatric patient, the infliximab half-life was 10.96 days which is in accordance with the reported values of 10.5 days [41] and 10.9 days [42]. The simulated trough concentration was 2.1 mg/L, while Adedokun et al. [43] reported a median of 1.9 (at week 30) for the 5 mg/kg intravenous dosing. Hence, it can be concluded that the PBPK simulation for a typical pediatric patent (14 years old, 60 kg) in Figure 7 was able to capture the general trend of the data, after the intravenous administration (5 mg/kg) of infliximab.

It is interesting to mention that in the trial by Adedokun et al. [43], there was no observed difference in infliximab pharmacokinetics between the stratified pediatric groups (6–12 vs. 12–17 years) with ulcerative colitis. Additionally, it was inferred that infliximab pharmacokinetics in pediatric patients were consistent with adult data, despite observing that the median serum infliximab concentrations were approximately 20% lower in children at various time points. However, it has been concluded that younger pediatric patients (median age 8) are underexposed with weight-based (mg/kg) intravenous dosing of infliximab compared to older pediatric patients (median age 14) [44]. In addition to this complex narrative, another study (C0168T72) [35] reported a higher percentage of participants in the younger age group discontinuing infliximab treatment (5 mg/kg) by week 8 due to non-response compared to those in the older age group. Nonetheless, the rates of discontinuation were similar after stratification in the maintenance phase [35]. Conclusively, these uncertainties underscore the complexity of treatment and disease dynamics and the need for further investigation to optimize the therapeutic and clinical outcomes with infliximab in pediatric (sub)populations with IBD.

The power of PBPK modeling can be leveraged also as a tool for model-informed dose suggestions in various clinical contexts. Hence, in the final two explorations, the PBPK framework was extended to these “what-if” scenarios in pediatric patients with IBD.

As already mentioned, there is no subcutaneous biobetter of infliximab intended for the pediatric population (the currently approved dose remains for adults only at a flat-fixed dose of 120 mg). Until now, there has been only one pediatric study [22] performed on seven individuals, that examined switching to the 120 mg infliximab dose in older (15–18 years) and heavier children (>50 kg, median 61 kg). In this trial, the median trough infliximab concentrations after switching were approximated to 14 mg/L (at 40 weeks post-switch). This concentration was also the upper limit of quantification, so it was not possible to accurately measure values above this threshold in four out of six children, clearly indicating that the true trough concentrations were higher with a flat-fixed 120 mg dosing [22].

The PBPK simulation of a typical older pediatric patient (14-year-old, 60 kg) (Figure 8) resulted in a trough infliximab concentration of 21 mg/L post-switching to 120 mg, which suggests that the PBPK model would be able to capture the higher true trend of the pediatric clinical trial data. Since there is no data for comparison at the moment, trough concentrations from the adult trial were used for visual inspection and for comparability. Namely, in Figure 8, a slightly higher trough concentration can be seen when compared to adults. However, this was not (for now) shown to be concerning in terms of safety [22]. Hence, it seems that the flat-fixed subcutaneous dose of 120 mg may be suitable (in terms of both effectiveness and safety) for older and heavier pediatric patients. Nonetheless, further clinical trials are necessary to comprehensively evaluate the advantages of subcutaneous administration and determine appropriate dose adjustments of infliximab in the pediatric population.

In the final exploration scenario (Figure 9), the developed PBPK model was applied to the virtual population. Similar to the approach for a typical virtual patient (adult or child with IBD), this approach provided insights into PBPK-model-informed dose suggestions for the target (sub)populations of interest.

Since infliximab is approved for treating IBD from the age of 6 and above, the target population (i.e., 150 pediatric individuals, 6–18 years of age) was stratified by weight [29] into three groups: 20–30 kg, 30–45 kg, and 45–70 kg. The choice of post-switch subcutaneous infliximab dose was guided by achieving comparable trough concentrations to those observed with the flat-fixed 120 mg dose in adults (Figure 9).

The PBPK-model-informed suggestion for pediatric patients weighing between 20 and 35 kg was 40 mg, with a slightly higher dose required to cover the entire prediction interval in this weight group (Figure 9, top). For patients weighing 35–45 kg, the suggested dose to cover the entire prediction was 80 mg (Figure 9, middle image), and for those weighing 45–70 kg, it was 120 mg (Figure 9, bottom image). The corresponding PBPK simulations show post-switch median trough concentrations around 14 mg/L, 18 mg/L, and 18.5 mg/L, respectively (with 5th–95th percentile intervals of 11.5–17 mg/L, 14.5–22 mg/L, and 16–23 mg/L).

Due to the slight differences in the reported trough concentrations after switching to subcutaneous infliximab, it is challenging to set a typical value for this exposure metric. Namely, the reported median trough concentrations, depending on the real-world trial in adults, ranged from 11 to 20 mg/L [37,39,45]. Based on that, it can be inferred that the PBPK predictions for the tested weight bands with the corresponding model-informed dose suggestions yielded exposure metrics comparable to those in adults receiving a flat-fixed 120 mg dose of subcutaneous infliximab. In addition, for the heaviest pediatric band (>45 kg) which corresponds to children older than 14 years of age, the prediction again confirms the suitability of administering a flat-fixed dose of 120 mg that is registered for adults.

It is also worth mentioning that the modeling assumption of the same bioavailability after subcutaneous administration of MAbs in younger children may also be debated, given the general anatomical differences in injection sites between children and adults [46]. These differences would imply, for example, variations in monoclonal antibody uptake, lymph flow, local degradation (catabolism), immunogenicity, and target-mediated drug disposition, which could be clinically relevant for the rate and extent of absorption among pediatric patients with IBD and contribute to a further increase in between-patient variability in pharmacokinetics, pharmacodynamics, and consequently, clinical outcomes [47]. One must also take into account the possible variability between pediatric patients and consequently modify the subcutaneous dose of infliximab to accommodate such variability if shown to be clinically relevant. Therefore, future clinical trials are anticipated to provide insights that can address the current uncertainties and further refine modeling approaches.

### 3.7. Limitations and Future Perspectives of the Study

In clinical settings, modeling and simulation approaches still face fragmentation and limited uptake. Nonetheless, there is a growing recognition of their significance, particularly in the realm of population pharmacokinetic and pharmacodynamic modeling.

PBPK modeling, while powerful, is inherently complex and demands expertise across multiple domains, including clinical pharmacology, pharmacokinetics, pharmacodynamics, physiology, and software proficiency. Due to its reliance on input parameters, the lack of comprehensive knowledge about them can undermine the reliability of predictions. PBPK models heavily rely on assumptions and approximations, posing challenges for model validation. This introduces uncertainty (error) into simulation predictions, further complicating the assessment of model reliability and its extrapolation abilities. In other words, the virtual human is as good as the real counterpart if the totality of accurate information is supported and undoubtedly confirmed by a fair amount of clinical evidence [8,13,48,49].

In this study, a more simplistic PBPK approach was employed, with some of the input parameters identified by an in-built software algorithm to match the observed clinical data. However, this common practice in modeling can introduce the likelihood of model/parameter identifiability issues, as the fitted values may not represent the true or experimentally determined values [48,49]. In other words, various parameter sets can generally yield similar fitting results. Additionally, apart from relying on robust clinical data, adjustments made to one parameter can cascade to others, ultimately affecting the overall parameter values of the model. However, in this study, the input parameters were very closely similar (or within the same order of magnitude) as the reported values in the literature, and as such, they enabled the PBPK model to capture general trends in the explored clinical scenarios.

Tailoring treatments with proper dosing options for individual patients is crucial for therapeutic success and personalized care. Extending the application of PBPK modeling beyond drug discovery and development into clinical settings could offer relevant information for clinicians. The future perspectives of the study involve comparing simulated scenarios with real-life clinical studies, while also encouraging drug manufacturers to assess their products’ effectiveness across all patient (sub)groups and advance the field of personalized medicine.

## 4. Conclusions

Utilizing state-of-the-art quantitative pharmacology tools represents the future approach for advancing pharmacological treatments in IBD. Hence, PBPK modeling, as a mechanistic framework, holds the capability to comprehensively incorporate both known and unknown disease mechanisms, as well as drug-specific characteristics. Leveraging this approach enables the exploration of novel strategies for more effective disease management.

PBPK modeling is commonly utilized in the drug development process. However, this study demonstrates its versatility in exploring diverse clinical scenarios related to infliximab use in the context of IBD. This includes investigating alternative dosing regimens and routes of administration across individuals and (sub)populations of clinical interest.

Finally, while simulations involving virtual individuals and populations must be complemented with real-life studies, the PBPK framework can also prompt drug manufacturers to reconsider the versatility of their products in effectively covering all patient groups.

## Figures and Tables

**Table 1:** Published clinical trial data on infliximab used for fitting [18] and model validation [14,17,20,21,22].

1. Clinical trial in healthy adults, single intravenous dose 5 mg/kg [18]
2. Clinical trial in adult patients with IBD [20] intravenous dose 5 mg/kg induction + maintenance up to week 54 subcutaneous switch, dose: 120 mg (skin of abdomen or upper thigh) subcutaneous switch, dose: 240 mg (skin of abdomen or upper thigh)
3. Clinical trial in adult patients with IBD, population pharmacokinetic study (subcutaneous switch to 120 mg in adults with IBD) and simulations on smaller, average, and heavier adult patients [17]
4. Clinical trial in pediatric population with IBD (intravenous route 5 mg/kg, ulcerative colitis) [14,21]
5. Clinical trial in older pediatric population with IBD (post-switch to subcutaneous 120 mg, skin of abdomen or upper thigh) [22]

**Table 2:** Input parameters for a baseline PBPK model in virtual healthy adults.

**TNF-α**
Reference concentration: 0.2 pm [16]
Localization: plasma and interstitial space; relative expression: 1 [14]
**Infliximab**
Molecular weight: 149.9 kDa [1]
Radius (solute): 5.18 nm [23]
Kd (FcRn) in endosomal space: 0.73 µmol/L [24]
Kd (FcRn) plasma/interstitial *: 4.54 mmol/L (i.e., negligible binding affinity [25])Kd (TNF-α) *: 120.17 pmol/L (100 pmol/L [26])
Koff (TNF-α) *: 1.331/ms (111/ms [27])
Kass (FcRn)*: 0.72 L/µmol/min (0.80 L/µmol/min [28])
**Anatomy and physiology of a virtual healthy adult**
Fraction endosomal: 0.20 [28]
Specific clearance in endosome *: 0.911/min
Fraction recycled to plasma: 1 [28]
Fraction of endosomal uptake from plasma *: 0.53
Rate constant for endosomal uptake: 0.291/min [28]
Rate constant for recycling from endosomal space *: 0.301/min
Kd (FcRn, endogenous IgG) in plasma/interstitial space: 10,000 µmol/L [28]
Kd (FcRn, endogenous IgG) in endosome: 0.63 µmol/L [28]
Kass (FcRn, endogenous IgG): 0.87 L/ µmol/min [28]

Fitted values are denoted by an asterisk (*), while values reported in the literature are shown in brackets. Kd (FcRn)—dissociation constant of infliximab for FcRn (neonatal fragment crystallizable receptor) in the endosomal space, or in the plasma/interstitial space (in neutral very weak affinity); Kd (TNF-α)—dissociation constant of infliximab for TNF-α (tumor necrosis factor alpha); Koff (TNF-α)—dissociation rate constant of infliximab for TNF-α; Kass (FcRn)—association constant of infliximab for FcRn; endogenous IgG—endogenous immunoglobulin G with its dissociation (Kd) and association (Kass) constant for FcRn.

## Data Availability

The original contributions presented in the study are included in the article/Supplementary Materials. Further inquiries can be directed to the corresponding authors.

## Supplementary Materials

The following supporting information on methodology can be downloaded at: https://www.mdpi.com/article/10.3390/biomedicines12091974/s1, Figure S1: Graphical representation of fold errors.
