# Supplementary material for: Infliximab in Inflammatory Bowel Disease: Leveraging Physiologically Based Pharmacokinetic Modeling in the Clinical Context
Source: Biomedicines. 2024 Sep 1;12(9):1974. doi: 10.3390/biomedicines12091974 (PMC11429320; doi:10.3390/biomedicines12091974)
Supplement: Supplementary file 1 [file biomedicines-12-01974-s001.zip › biomedicines-3147184-supplementary.pdf]

Supplementary Materials

The model for protein drugs and large molecules extends to transcapillary drug exchange (described by the two-pore formalism) [14], lymphatic flows, and endosomal space, which includes drug degradation and protection from degradation facilitated by the neonatal fragment crystallizable receptor (FcRn) (i.e., FcRn submodel) [9]. The portion of the drug bound to FcRn within the endosomal space is recycled back to the plasma and the interstitial space, while the portion not bound to FcRn is cleared from the endosomal space. Within the submodel, the presence of naturally occurring antibodies (e.g., IgG) in the human body is also taken into account, since their competition for FcRn receptors can influence the pharmacokinetics of the drug, affecting drug distribution and clearance [9].

It is also important to note that drug-specific parameters important for PBPK simulation differ between small molecule drugs and proteins, and only a few inputs are required for model setup. It is crucial to deselect the "small molecule" option in the compound building block. If this option remains selected, the processes relevant to the drug of interest (in this case MAb), such as entry into the endosomal space, will not be applicable.

The baseline model (initially developed for a typical healthy adult) was subsequently extended to include IBD patients (both adults and pediatric). This extension aimed to reflect the typical (average) pharmacokinetic and biometric profile of the population derived from corresponding clinical trials, and/or to explore various “what-if” scenarios. When the PBPK simulation predictions were comparable with the overlaid mean concentration-time profiles of the individuals from the real-world clinical trials, it was assumed that the model is able to capture the overall trend. The validation of model performance relied on comparisons with both available trial data and relevant publications, including exposure metrics, pharmacokinetic parameter values and visual inspection. Validation per se can also be observed in the seamless transition from one exploration scenario to another, encompassing various patients and populations with IBD, where the PBPK predictions aligned with real-world information derived from the corresponding drug's concentration-time profile.

As stated earlier, MAbs within cellular endosomes can be degraded (i.e., cleared) or protected from being broken down by binding to the FcRn receptor. To differentiate a healthy individual from a patient with IBD in the (patho)physiological context, the endosomal clearance value was manually adapted (i.e., increased) after initial fitting. This adjustment served as a lumped surrogate for factors affecting infliximab exposure and its disposition, i.e., increased clearance. Additionally, it mimicked the unwanted effects of gut inflammation, immunogenicity (anti-drug antibodies), and leaky gut on the infliximab disposition [1]. By integrating both immune- and non-immune-mediated infliximab clearance mechanisms, this simplistic approach provides pharmacokinetic profiles that correspond to moderate and severe IBD in real-life clinical setting.

Similar methodologies, such as depicting patient profiles in the (patho)physiological context of IBD using an average patient from a trial and manual adaptation of infliximab clearance, have been employed separately in other publications [13,18].

Fold error (FE), defined as the ratio of the simulated value to the observed value, is used by some researchers to assess model performance (Supplement Figure 1.). FE quantifies how much the prediction deviates from the actual observed value. A FE value of 1, represented by the blue line in the figure, indicates an ideal scenario where the prediction exactly matches the observed value. Based on the FE values of the analyzed PK parameters and exposure metrics, it can be inferred that the outcomes of the simulated clinical scenarios are close to the real-world data. Perfect alignment is not expected due to inherent uncertainties, significant interpatient variability (studied cohort), and the evolving nature of the disease (i.e., disease remission vs. relapse), which collectively influence the overall values. In addition, the 2-fold error criterion is also used by some researchers to assess the accuracy of a model's predictions by checking if the predicted value is within a factor of 2 of the observed value. If the ratio of the simulated to observed value is within the range of 0.5 to 2.0, it indicates that the model's predictions are reasonably close to the actual observed data.

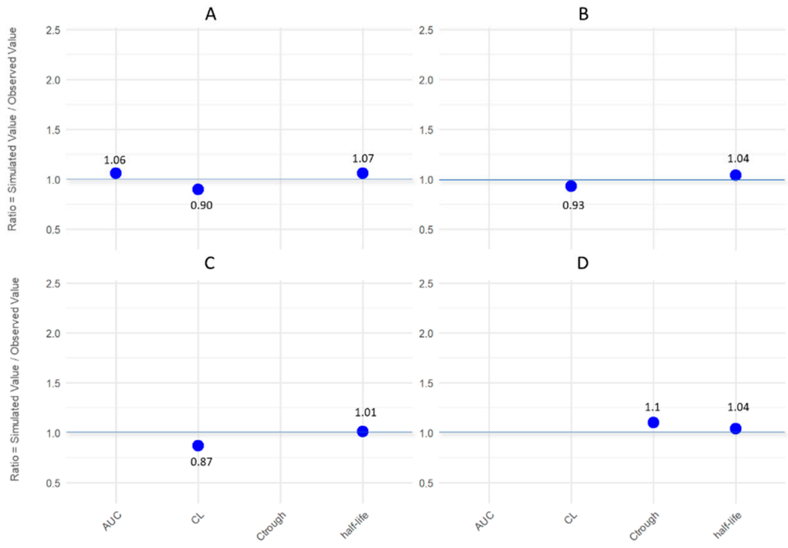

Supplement Figure S1. Graphical representation of fold errors (i.e., ratios of simulated values to observed values) depicted as blue dots. The plot includes PK parameters [Clearance (CL) and half-life], as well as exposure metrics [Area Under the Curve (AUC) and Ctrough], across simulated scenarios. Part A corresponds to the simulated predictions shown in Figure 4 [17,30], Part B to those in Figure 5 [32], Part C to Figure 6A [16], and Part D to Figure 7 [41,43]. The ideal fit is represented by the blue line.

The reference numbers in the Supplement correspond directly to the main references listed in the paper.
